# BioWMS: a web-based Workflow Management System for bioinformatics

**DOI:** 10.1186/1471-2105-8-S1-S2

**Published:** 2007-03-08

**Authors:** Ezio Bartocci, Flavio Corradini, Emanuela Merelli, Lorenzo Scortichini

**Affiliations:** 1 Dipartimento di Matematica e Informatica, Università di Camerino, Via Madonna delle Carceri n.9, Camerino(MC), I-62032, Italy

## Abstract

**Background:**

An in-silico experiment can be naturally specified as a workflow of activities implementing, in a standardized environment, the process of data and control analysis. A workflow has the advantage to be reproducible, traceable and compositional by reusing other workflows. In order to support the daily work of a bioscientist, several Workflow Management Systems (WMSs) have been proposed in bioinformatics. Generally, these systems centralize the workflow enactment and do not exploit standard process definition languages to describe, in order to be reusable, workflows. While almost all WMSs require heavy stand-alone applications to specify new workflows, only few of them provide a web-based process definition tool.

**Results:**

We have developed BioWMS, a Workflow Management System that supports, through a web-based interface, the definition, the execution and the results management of an in-silico experiment. BioWMS has been implemented over an agent-based middleware. It dynamically generates, from a user workflow specification, a domain-specific, agent-based workflow engine. Our approach exploits the proactiveness and mobility of the agent-based technology to embed, inside agents behaviour, the application domain features. Agents are workflow executors and the resulting workflow engine is a multiagent system – a distributed, concurrent system – typically open, flexible, and adaptative. A demo is available at .

**Conclusion:**

BioWMS, supported by Hermes mobile computing middleware, guarantees the flexibility, scalability and fault tolerance required to a workflow enactment over distributed and heterogeneous environment. BioWMS is funded by the FIRB project LITBIO (Laboratory for Interdisciplinary Technologies in Bioinformatics).

## Background

Over the past few years, new high-throughput methods for data collection in life science have greatly increased data generation. Parallel to the rise of biological databases, many bioinformatics tools, to query and analyze biological data, have been made available. As consequence, the traditional scientific process has become computationally intensive and in-silico experiments – described as processes of activities to test hypotheses, derive a summary and search for patterns [1] – are labouriously executed in a large, distributed and dynamic environment.

Since the execution of an in-silico experiment may simultaneously demand data integration from several application domains (e.g. biology, biochemistry, oncology), tool integration – analysis techniques (e.g. data mining and text mining) computational methods often offered as services that are dynamically updated, added or removed [2] –, it can be naturally specified as workflows of activities that implement data and control analysis processes in standardized but dynamic environments. The workflow has the advantage to be reproducible, traceable and to reuse intermediate results; fundamental features to validate a scientific experiment.

The software component that "defines, manages and executes workflows through the execution of software whose order of execution is driven by a computer representation of the workflow logic", according to Workflow Management Coalition (WfMC) Reference Model [3], is named Workflow Management System (WMS). In bioinformatics, several WMSs-like [4-7] have been already developed and adopted to support the daily work of a bioscientist.

Taverna [4] – a part of MyGrid project [8] – has mainly the aim to integrate Web Services by workflows specified in a choreography language: *XML Simple conceptual unified flow language *(XScufl). Being Taverna editor embedded with its engine in a Java stand-alone application, it is quite heavy, for an end-user, to download it.

Biopipe [5] framework, instead, provides a set of wrappers to directly interface resources like executable programs and data adaptors. It does not support the use of synchronization operators, like fork and join, because a bioinformatics experiment is considered just a sequential pipeline.

Wildfire [7] is another WMS that provides an integrated environment for the construction and execution of workflows based only on Jemboss [9] applications.

Pegasys [6] system enables bioscientists to create and manage sequence analysis workflows. It includes numerous analytical tools and provides database capacities to maximize information captured during the execution of a workflow.

All the above mentioned WMSs describe, often with different expressive power, an in-silico experiment without using standard workflow specification languages. Only recently, Garcia, in [10], has proposed a meta-analysis of syntactic components and algebraic operators capable of representing analytical workflows in bioinformatics. Remarkable are also standard process definition languages – i.e XPDL [11], BPEL [12] – are well-studied from the *control-flow *perspective; they allow to describe activities and their execution ordering through different constructs as *sequence*, *choice*, *parallelism*, *join synchronization*, etc.. Standard languages are also equipped of several tools to graphically edit workflow specifications, characteristic that is useful for a user not expert in programming.

Another significant feature that characterizes most of WMSs is their client/server architectural style, in which the workflow enactment is entrusted to a central component, that acts as a server and is responsible for the correct workflow execution. A monolithic architecture, as that of above mentioned WMSs, does not allow the execution of workflow, or parts of it, over a distributed and heterogeneous environments due to lack of flexibility, scalability and fault tolerance, features required for a distributed cross-organizational workflow.

An attempt to overcome these limitations, has been proposed by Cichocki in [13] in the Migrating Workflow Model (MWM). In this model, instances of a workflow or parts of it can migrate among sites participating in the workflow execution; it means, the code and the whole execution state, including all data gathered during the execution, might be transferred to another site. This model provides two main benefits: the first regards the migrating workflow with respect to the decrease of traffic network; often it is less heavy to transfer the code implementing workflow specification than the amount of data needed during its execution. The second asset concerns the possibility for the workflow to be executed even in mobile and weekly connected network of devices. But, the MWM requires a suitable middleware that guarantees code mobility support.

In this paper, we present BioWMS, a Workflow Management System which, from the one hand, provides a web-based user interface for the definition, the execution and the results management of an in-silico experiment and from the other, it exploits the agent-oriented technology to implement a Migrating Workflow Model. BioWMS dynamically generates the workflow engine associated with a single user workflow specification. The agent-based technology allows to embed application domain features inside the agents knowledge and proactiveness and mobility inside the agent behaviour. The resulting workflow engine is a multiagent system – a distributed, concurrent system – typically open, flexible, adaptive and mobile.

## Results and Discussion

**Figure 1:**
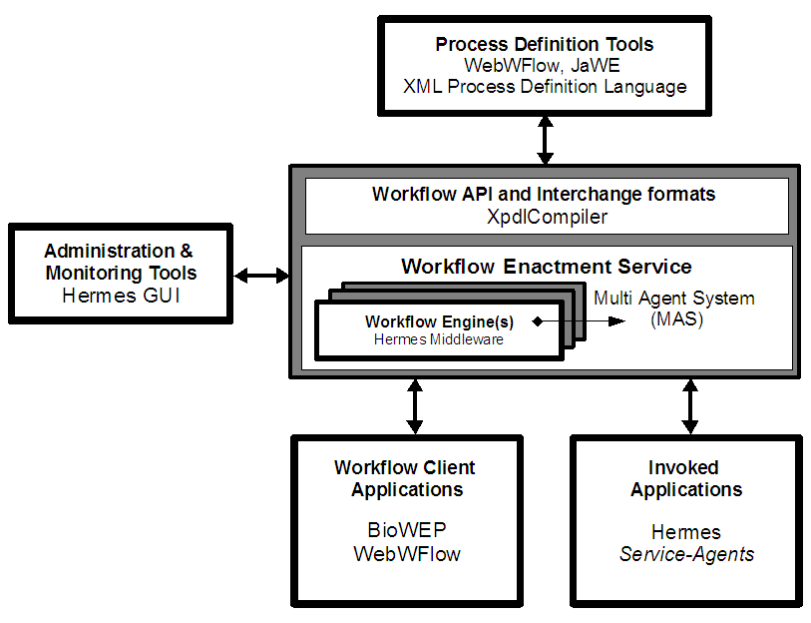
**BioWMS according to WfMC reference model**. The WfMC Reference Model specifies a framework for workflow systems, identifying their characteristics, functions and interfaces.

The Workflow Management Coalition (WfMC) defines a WMS as a "system that defines, creates and manages the execution of workflows through the use of software, running on one or more workflow engines, which is able to interpret the process definition, interact with workflow participants and, where required, invoke the use of IT tools and applications" [3]. According to this definition we have developed BioWMS as an automated framework to define, create and manage the execution of an in-silico experiment defined as a workflow of user domain activities. Figure 1 shows BioWMS according to the Workflow Reference Model introduced by the WfMC in [3].

*Process definition tools *are used to create the process description of a workflow in a computer processable form. The workflow editor is the program that supports the workflow specification by composing activities in a graphical environment. BioWMS provides a web-based editor, called WebWFlow, that enables the definifion of a workflow in the XML Process Definition Language (XPDL) [11], a WfMC standard. As a consequence, the workflow specification can be edited also by other applications compliant to this standard as for example the JaWE [14] editor.

*Workflow client applications *are used to execute existing or previous saved workflows, to check the workflow execution state and to manage the produced results. WebWFlow, the web-based editor of BioWMS, provides also these facilities. Another workflow client application developed for BioWMS is BioWEP [15], a web portal suitable for the ontology-based selection and enactment of predefined and annotated workflows.

The *workflow enactment service *provides the run-time environment in which process instantiation and activation occur, utilising one or more workflow management engines, responsible for interpreting and activating part, or all, of the process definition and interacting with the external resources -*invoked applications*- necessary to process the various activities. The workflow enactment in BioWMS is supported by the Hermes [16] agent-based middleware. The XpdlCompiler is an Hermes special component that generates a mobile multiagent system – workflow executors – from the user workflow specification. The workflow specification is the coordination model that describes how the generated agents cooperate with each other to reach a particular goal. The middleware provides the necessary software infrastructure that allows the migration of the workflow executors to different sites transparently to users.

The Hermes Graphical User Interface (GUI) is an *administration and monitoring tool *that allows checking, at any time, of the computational resources available, the memory consumption and the agents that are running.

### An example of workflow in BioWMS

**Figure 2:**
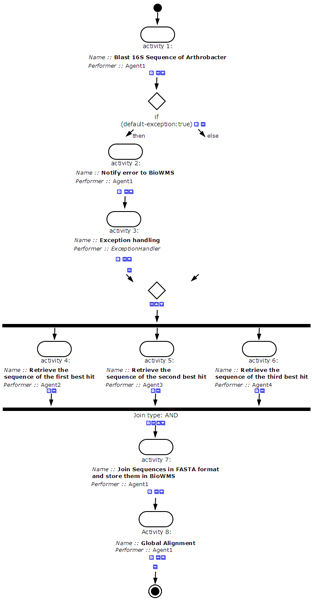
**Example of a workflow in BioWMS**. Screenshot of a workflow example in BioWMS. The goal is to obtain a global alignment of the Arthrobacter koreensis partial 16S rRNA gene sequence [GI:70663476] with the first three best hits of a BLAST [17] alignment in the DDBJ [18] database.

In this section we provide an example of workflow in BioWMS. The goal is to obtain a global alignment of the Arthrobacter koreensis partial 16S rRNA gene sequence [GI:70663476] with the first three best hits of a BLAST [17] alignment in the DDBJ [18] database. Figure 2 shows how the workflow appears in BioWMS. BioWMS provides a repository of user domain activities that can be customizable by configuring properly their parameters. As first activity we choose one that receives as parameters a sequence and other blast parameters and gets the best hits using the BLAST alignment. If an exception is thrown during the execution of this activity we want to notify the error to BioWMS – activity 2 – to handle the errors – activity 3 – and to proceed in the workflow execution, else we retrieve concurrently – activities 4,5 and 6 – the sequences of the three best hits obtained. At the end of all of these three activities we want to join the sequences and store them – activity 7 – into BioWMS in a FASTA [19] representation.

To get the global alignment we choose an activity – activity 8 – that receives as parameter a list of sequences in FASTA and calculates the global alignment using ClustalW [20] tool. For further details of this example we provide in [21] a videoclip of the workflow definition and execution phases in BioWMS.

### Designing a workflow

The activities used in a workflow can be configured with several parameters, in this way it is possible to reuse the same activity for different workflows. In the previous example, the BLAST activity takes in input a sequence and blast parameters such as the blast tool – e.g. blastn – and the database – e.g. ddbj – to choose. This activity can be re-used in each workflow that needs a local alignment of a particular sequence with others in a specific database.

**Figure 3:**
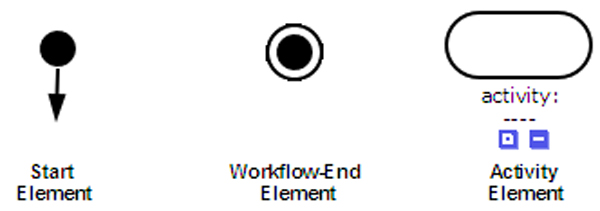
**Graphical notation of Workflow elements available in WebWFlow**. BioWMS has three main elements: 1) Workflow-Start element; 2) Workflow-Stop element; 3) Workflow-Activity element.

Any workflow can be specified by using the graphical notation offered by the WebWFlow the BioWMS editor. A workflow can be started and ended by special graphical elements those shown in Figure 3, in particular, the *Workflow end *element is the point in which the workflow has to be terminated. In the same figure is shown the graphical notation of an activity element.

**Figure 4:**
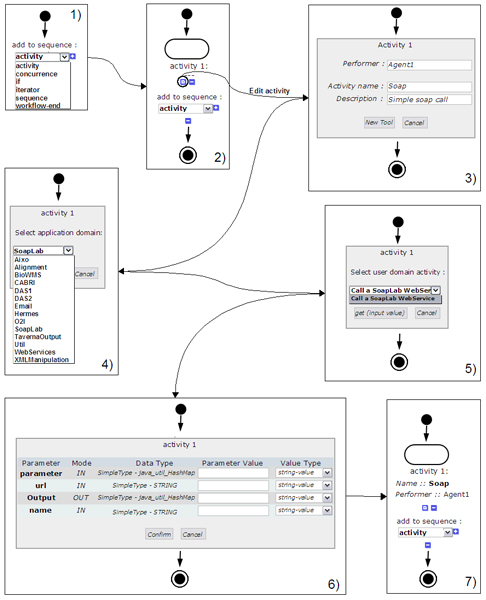
**Setting up an activity: step-by-step**. Steps required to add and set a domain specific activity: 1) Select the *activity *item from the multiple choice box; 2) Edit the added activity; 3) Specify the activity performer; 4) Select the activity application domain; 5) Select the user domain activity; 6) Fill the input/output formal parameters; 7) The activity is added and set.

**Figure 5:**
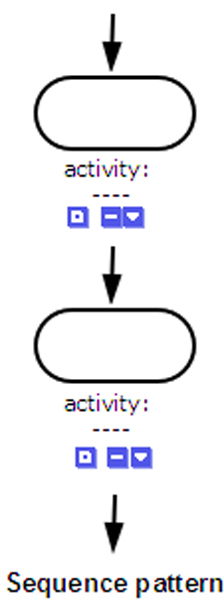
Sequence Workflow patterns available using WebWFlow.

WebWFlow, the BioWMS editor, enables the composition of both primitive and complex activities. A primitive activity is an activity that can be directly executed. A complex activity is a user activity that must be specified before it can be used; the specification of a complex activity is a workflow of complex and simple activities. By using complex activities the specification of workflows is simplified because they enhance both hierarchical specification and reuse: we can use an already existing complex activity without caring of its specification. In the previous example, we can use the *BLAST activity*, without knowing that it relies on the *CallWebService *primitive activity that in turns it collects the results from BLAST. In this case, user can customize BLAST activity without knowing what is a WebService. Users can use complex activities and stored workflows to increase productivity when specifying new workflows. Moreover, large libraries of both domain specific primitives and complex activities can be loaded to specialize the editor for a specific application domain. Figure 4 shows the steps required by WebWFlow to add and customize an activity and Table 1 shows the libraries of domain-specific activities actually available in BioWMS. A user can specify the order of activities execution using special control-flow patterns. The *Sequence *pattern, shown in Figure 5, allows to execute an activity after another. It is generally used when the output of an activity must be piped as input of the subsequent. In the workflow example in Figure 2, the sequences in FASTA format obtained by activity 7 are piped as input of the activity 8 that performs the global alignment.

**Figure 6:**
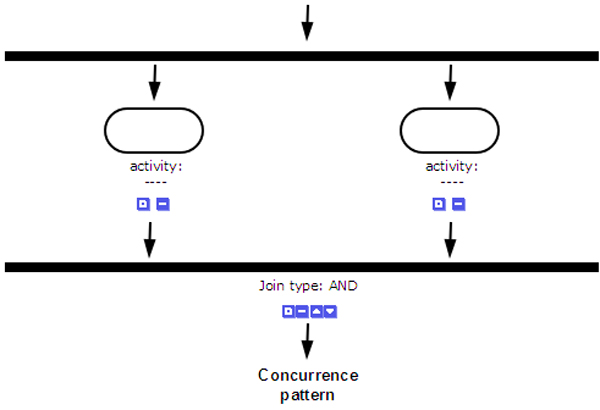
Concurrence Workflow patterns available using WebWFlow.

Figure 6 shows the *Concurrence *pattern that enables the parallel execution of two or more activities. It is possible to assign each of these concurrent activities to different performers corresponding to the Workflow Executors. In this case, the code mobility, supported by Hermes middleware, can be exploited to improve the computational load balancing of a workflow. In the workflow example we use this pattern to retrieve concurrently -activities 4,5 and 6- the sequences of the best hits.

**Figure 7:**
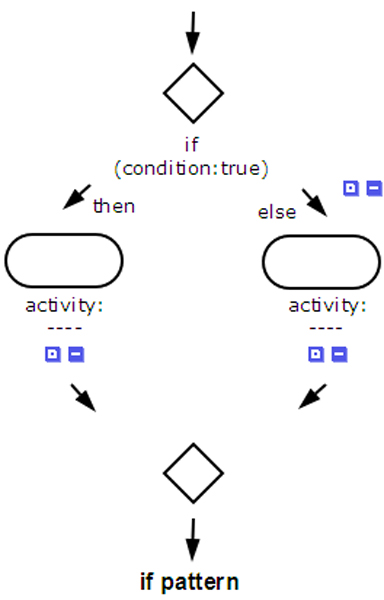
If Workflow patterns available using WebWFlow.

Figure 7 shows the *If *pattern that defines a conditional routing where the choice of the activity to be executed is case-driven. An error or exception can be caught and considered as special case, so that the workflow becomes fault tolerant and the execution can select an alternative path when something goes wrong. In the example in Figure 2 we use *if *pattern to have a decision point when activity 1 generates or not an exception.

**Figure 8:**
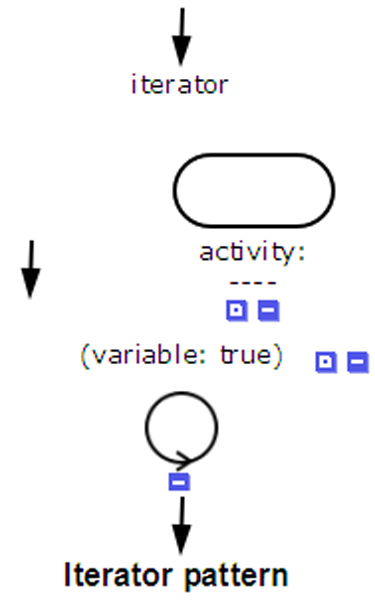
Iterator Workflow patterns available using WebWFlow.

**Figure 9:**
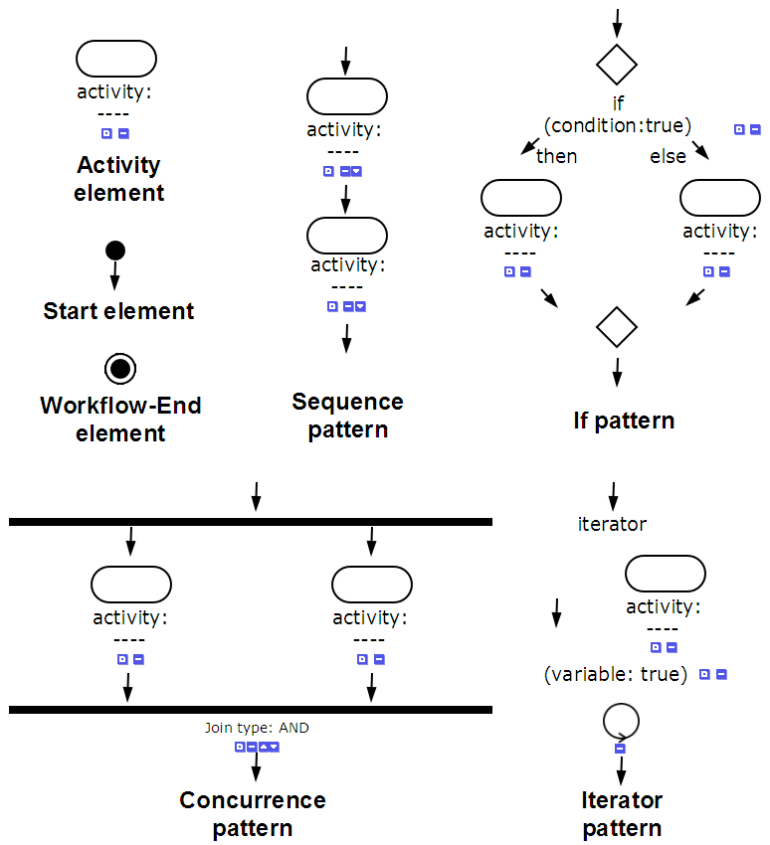
**Workflow patterns available using WebWFlow**. The basic workflow elements are: workflow-start element, workflow-end element and activity element. The control-flow patterns available in WebWFlow are: *sequence*, *concurrence*, *if *and *iterator*.

The *Iteration*, whose graphical notation is shown in Figure 8, is a pattern enabling the cyclic execution of one or more activities. When a special case occurs the control-flow leaves the cycle and the workflow execution continues. The arrow on the left hand side of the graphical notation addresses the direction of the execution. All possible workflow patterns and elements allowed in BioWMS by using WebWFlow editor are summarized in Figure 9.

### Loading and executing a workflow

Workflows stored in BioWMS can be *public *or *private*. While a public workflow is provided by BioWMS and is visible to all users, a private one is available only for the user that has previously defined it. The web-interface allows to view workflow both with a graphical notation and with the corresponding XPDL workflow specification. After a user has defined its workflow and has submitted it, BioWMS compiles it in a multiagent system by exploiting the agent-based middleware. BioWMS supports long-running activities and the execution is independent from the user control.

### Managing results

**Figure 10:**
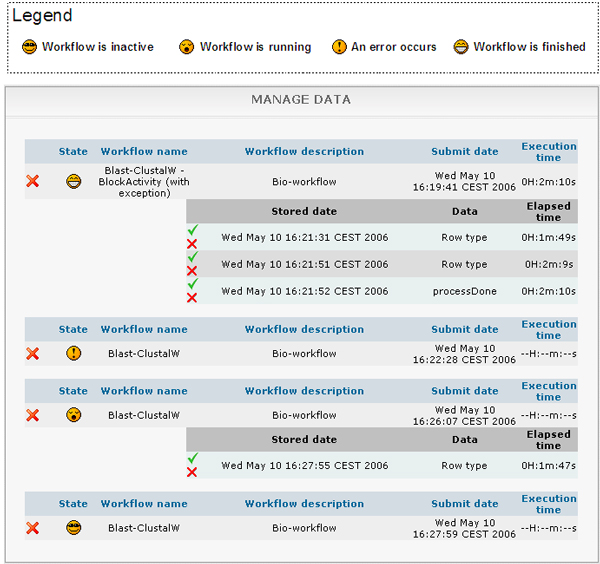
**Managing results**. User can check at any time the state of the submitted workflows and the corresponding produced results. For each workflow is provided a measure of its execution time.

At any time users can check the workflow execution state and the partial produced results. Figure 10 shows a screenshot of "manage data" web page. This interface provides the elapsed time for each workflow execution. Partial results can be stored during the enactment in several formats applying XSLT [22] stylesheets or using Taverna format. To do this, user has to choose, during the workflow definition phase, the domain-specific activities from the "XML manipulation" or "Taverna Output" libraries.

### Conclusions and future work

We have developed BioWMS, a Workflow Management System for the design of an activity-based in-silico experiment. BioWMS, provides a web-based user editor interface and is supported by mobile agent-based technology, it guarantees the flexibility, scalability and fault tolerance required for the workflow enactment over distributed and heterogeneous systems.

As a future work we aim to extend BioWMS with a component called Resourceome [23] on which we are in parallel working on. Resourceome is a system that keeps "alive" an index of resources in the bioinformatics domain using a specific ontology of resource information. The Resourceome directly assists scientists in the hard navigation in the ocean of bioinformatics resources. The integration of BioWMS with Resourceome might support the final user both in selecting the most suitable activity and to setting-up a new one. The Resourceome itself would dynamically support workflow enactment, providing all the required resources available at runtime. We believe that the workflow technology together with an effective and effcient resource management system could be a good start to face the complexity that surrounds scientist's work and then to help him/her in taking advantage of the huge amount of available resources.

## Methods

A workflow is a distributed application that involves the coordinated execution of human and system activities, usually, in an heterogeneous environment. We consider a workflow as coordination model for a pool of mobile agents -workflow executors- that implements the workflow engine for a specific workflow instance. In this context agents are autonomous active entities, encapsulate the execution of independent activities and execute their tasks concurrently to the work of the other agents. A collection of agents able to cooperate, in their autonomy, for a common goal forms a multiagent system (MAS) [24].

### Workflow compilation

In our approach, the generation of the workflow engine is performed by a compiler [16] in a two phase agent-generation procedure. In the first step a User Level Workflow (ULW), specified by a workflow specification language, is mapped to an Agent Level Workflow (ALW). This mapping is performed by recursively substituting activities of the user-level specification with a workflow of primitive agent-level activities. A User-Level Activity Database (ULAD) maintains the correspondence between user-level activities and ALW. The ALW specifies all entities involved in the execution of a workflow; thus the constraint of spatial and temporal coupling communication can be respected since the compiler knows exactly when communication takes place and which are the receivers and which the senders. In the second step, the compiler concretely generates agents from the ALW specification. To achieve this result, the compiler uses the User- Level Activity Implementation Database (ULAID) and the Database of Skeleton (DoS). The ULAID stores the implementation of agent-level activities, and DoS stores empty implementation of agents (the skeletons). A WE is obtained by plugging the specific behavior into the skeleton. The resulting set of WEs gives rise to an agent-based workflow engine whose role will be compliant to the WMS architecture as before described in Figure 1. The above approach has been implemented on Hermes [16] architecture whose detailed description is given in the next section.

### Hermes architecture

Hermes [16,25] is an agent-based mobile middleware, for the design and execution of activity-based applications in distributed environments. It is structured as a component-based, agent-oriented system with a 3-layer software architecture: user layer, system layer and run-time layer. Each layer is customizable and is independent from the others.

**Figure 11:**
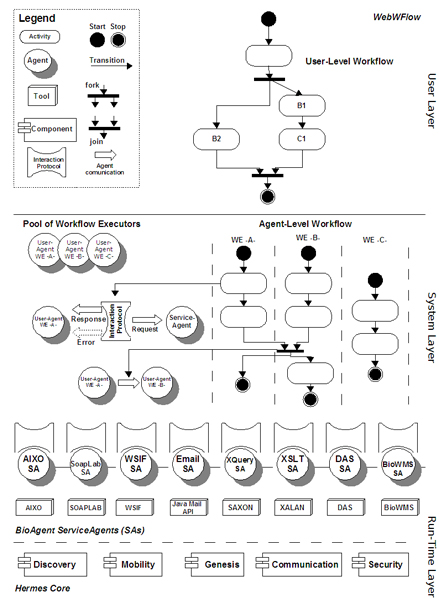
**BioWMS has been implemented over Hermes middleware**. Entities located at each layer of Hermes Architecture. Any user level workflow is mapped into an agent-level workflow and compiled to a pool of mobile user agents, the Workflow Executors (WEs) which interact with Service Agents (SAs).

BioWMS is a distributed oriented application that relies on Hermes middleware architecture as Figure 11 shows. User layer allows designers to specify their application as a workflow of activities using the graphical notation provided by WebWFlow. System layer provides a context-aware compiler to generate a pool of user mobile agents from the workflow specification. Run-time layer supports the activation of a set of specialized service agents, and it provides all necessary components to allow agent discovery, mobility, creation, communication and security. Service-Agents (SAs) in the run-time layer are localized to one platform to interface with the local execution environment. User-Agents (UAs) in the system layer are Workflow Executors (WEs), created for a specific goal that, in theory, can be reached in a finite time by interacting with other agents; afterward the agent will die by killing itself. At the end of a two steps process a compiler translates XPDL to a Multi Agent System (MAS) as Java bytecode ready to be executed in Hermes middleware.

Hermes can be configured for specific application domains by adding domain-specific component libraries and thus customizing in a proper way through service agents. It represents a flexible environment suitably designed to support the bioscientist's activities during an in-silico experiment. The main functionalities of BioWMS are provided by Hermes through a set of cooperative bio-service agents (SA). These are shown in Figure 11 and are described as follows:

#### Data and Tools Integration

AIXO [26] SA provides a set of wrappers able to access and to present any data source as a collection of XML documents. AIXO (Any Input XML Output) is flexible and modular, it allows to manage many input data sources from HTML to XML, database, flat file, CGI and command line programs;

#### Web Services

WSIF SA Service Agent allows other agents to dynamically invoke a Web Service.

Moreover, SoapLab SA [27] can control a set of Web Services providing programmatic access to many bioinformatics applications on remote computers;

#### XML Manipulation

XQuery [28] and XSLT [22] SAs provide tools to transform, manipulate and query an XML document creating a suitable view of a Web Service invocation or a wrapper output;

#### Distributed Annotation System (DAS)

DAS SA enables the access to DAS [29] sources;

#### Input and Output Management

BioWMS SA allows the storage of partial results during the execution. Furthermore, Email SA allows user to receive the final and intermediate results by email.

## Authors' contributions

EB wrote the paper and participated in tool design and in its development. LS implemented the Web-based interface of BioWMS. EM and FC conceived the study, participated and coordinated the paper and tool design, and helped to draft the manuscript. All authors read and approved the final manuscript.

## Figures and Tables

**Table 1:** Application domain libraries available

**Application domain library**	**Description**
AIXO	A set of primitive activities to convert a format to XML
Alignment	A set of primitive and complex activities for sequence alignment
BioWMS	A set of primitive activities to store and to log messages in BioWMS
Email	A set of primitive activities to send emails
Hermes	A set of primitive activities to exploit Hermes middleware
O2I	A set of complex activities wrapping O2I SoapLab services
Taverna Output	A set of primitive activities to store data in Taverna format
Util	A set of primitive and complex activities to manage data
WebServices	A set of primitive activities to invoke a Web Service
XML Manipulation	A set of primitive and complex activities using XSLT and XQuery

## References

[B1] Stevens R, Glover K, Greenhalgh C, Jennings C, Pearce S, Li P, Radenkovic M, Wipat A, Cox SJ (2003). Performing in silico experiments on the Grid: a users perspective. Proceedings of UK e-Science All Hands Meeting: 2–4 September 2003; Nottingham.

[B2] Stein L (2002). Creating a bioinformatics nation. Nature.

[B3] Hollinsworth D (1994). The Workflow Reference Model. Tech Rep TC00-Workflow Management Coalition.

[B4] Oinn T, Addis M, Ferris J, Marvin D, Senger M, Greenwood M, Carver T, Glover K, Pocock M, Wipat A, Li P (2004). Taverna: a tool for the composition and enactment of bioinformatics workflows. Bioinformatics.

[B5] Hoon S, Ratnapu K, Chia J, Kumarasamy B, Juguang X, Clamp M, Stabenau A, Potter S, Clarke L, Stupka E (2003). Biopipe: a flexible framework for protocol-based bioinformatics analysis. Genome Res.

[B6] Shah S, He D, Sawkins J, Druce J, Quon G, Lett D, Zheng G, Xu T, Ouellette B (2004). Pegasys: software for executing and integrating analyses of biological sequences. BMC Bioinformatics.

[B7] Tang F, Chua C, Ho L, Lim Y, Issac P, Krishnan A (2005). Wildfire: distributed, Grid-enabled workflow construction and execution. BMC Bioinformatics.

[B8] Stevens R, Robinson A, Goble C (2003). myGrid: personalised bioinformatics on the information grid. Bioinformatics.

[B9] Carver T, Bleasby A (2003). The design of Jemboss: a graphical user interface to EMBOSS. Bioinformatics.

[B10] Garcia Castro A, Thoraval S, Garcia L, Ragan M (2005). Workflows in bioinformatics: meta-analysis and prototype implementation of a workflow generator. BMC Bioinformatics.

[B11] XML Process Definition Language. http://xml.coverpages.org/XPDL20010522.pdf.

[B12] OASIS WSBPEL Specification Draft. http://docs.oasis-open.org/wsbpel/2.0/wsbpel-v20-rddl.html.

[B13] Cichocki A, Rusinkiewicz M (2004). Providing Transactional Properties for Migrating Workflows. MONET.

[B14] Enhydra JaWE. http://www.enhydra.org/workflow/jawe/index.html.

[B15] Romano P, Bartocci E, Bertolini G, De Paoli F, Marra D, Mauri G, Merelli E, Milanesi L (2006). Biowep: a workflow enactment portal for bioinformatics applications. BMC Bioinformatics.

[B16] Corradini F, Merelli E (2005). Hermes: agent-base middleware for mobile computing. Mobile Computing, LNCS.

[B17] Ye J, McGinnis S, Madden T (2006). BLAST: improvements for better sequence analysis. Nucleic Acids Res.

[B18] DNA Data Bank of Japan. http://www.ddbj.nig.ac.jp/.

[B19] FASTA format. http://en.wikipedia.org/wiki/Fasta_format.

[B20] Thompson J, Higgins D, Gibson T (1994). CLUSTAL W: improving the sensitivity of progressive multiple sequence alignment through sequence weighting, position-specific gap penalties and weight matrix choice. Nucleic Acids Res.

[B21] A flash presentation of BioWMS. http://litbio.cs.unicam.it/biowms/video.html.

[B22] XSLT: XSL Transformations. http://www.w3.org/TR/xslt.

[B23] Cannata N, Merelli E, Altman R (2005). Time to Organize the Bioinformatics Resourceome. PloS Computational Biology.

[B24] Jennings N, R N (2000). On Agent based Software Engineering. Artificial Intelligence.

[B25] Site of HermesV2. http://hermes.cs.unicam.it/.

[B26] Bartocci E, Mariani L, Merelli E (2003). An XML view of the "World". ICEIS (1).

[B27] Senger M, Rice P, Oinn T, Cox SJ (2003). Soaplab – a unified Sesame door to analysis tools pages. Proceedings of UK e-Science All Hands Meeting: 2–4 September 2003; Nottingham.

[B28] XQuery 1.0: An XML Query Language. http://www.w3.org/TR/xquery/.

[B29] Dowell R, Jokerst R, Day A, Eddy S, Stein L (2001). The distributed annotation system. BMC Bioinformatics.

